# Gene Expression Profiling of Bronchoalveolar Lavage Cells Preceding a Clinical Diagnosis of Chronic Lung Allograft Dysfunction

**DOI:** 10.1371/journal.pone.0169894

**Published:** 2017-01-19

**Authors:** S. Samuel Weigt, Xiaoyan Wang, Vyacheslav Palchevskiy, Aric L. Gregson, Naman Patel, Ariss DerHovanessian, Michael Y. Shino, David M. Sayah, Shirin Birjandi, Joseph P. Lynch, Rajan Saggar, Abbas Ardehali, David J. Ross, Scott M. Palmer, David Elashoff, John A. Belperio

**Affiliations:** 1 Department of Medicine, David Geffen School of Medicine, University of California, Los Angeles, California, United States of America; 2 Department of Surgery, David Geffen School of Medicine, University of California, Los Angeles, California, United States of America; 3 Department of Medicine, Duke University, Durham, North Carolina, United States of America; University of Toledo, UNITED STATES

## Abstract

**Background:**

Chronic Lung Allograft Dysfunction (CLAD) is the main limitation to long-term survival after lung transplantation. Although CLAD is usually not responsive to treatment, earlier identification may improve treatment prospects.

**Methods:**

In a nested case control study, 1-year post transplant surveillance bronchoalveolar lavage (BAL) fluid samples were obtained from incipient CLAD (n = 9) and CLAD free (n = 8) lung transplant recipients. Incipient CLAD cases were diagnosed with CLAD within 2 years, while controls were free from CLAD for at least 4 years following bronchoscopy. Transcription profiles in the BAL cell pellets were assayed with the HG-U133 Plus 2.0 microarray (Affymetrix). Differential gene expression analysis, based on an absolute fold change (incipient CLAD vs no CLAD) >2.0 and an unadjusted p-value ≤0.05, generated a candidate list containing 55 differentially expressed probe sets (51 up-regulated, 4 down-regulated).

**Results:**

The cell pellets in incipient CLAD cases were skewed toward immune response pathways, dominated by genes related to recruitment, retention, activation and proliferation of cytotoxic lymphocytes (CD8^+^ T-cells and natural killer cells). Both hierarchical clustering and a supervised machine learning tool were able to correctly categorize most samples (82.3% and 94.1% respectively) into incipient CLAD and CLAD-free categories.

**Conclusions:**

These findings suggest that a pathobiology, similar to AR, precedes a clinical diagnosis of CLAD. A larger prospective investigation of the BAL cell pellet transcriptome as a biomarker for CLAD risk stratification is warranted.

## Introduction

Lung transplant is a therapeutic option for end-stage pulmonary disorders, but long-term survival is dependent upon remaining free from chronic lung allograft dysfunction (CLAD), which affects greater than 50% of recipients within 5 years. CLAD is characterized by the inexorable loss of lung function, and the typical survival following CLAD diagnosis is less than 3 years [1]. The diagnosis of CLAD relies on a 20% or greater decline in the forced expiratory volume in 1 second (FEV_1_), sustained over at least 3 weeks, from the post-transplant baseline. Although several phenotypes of CLAD have been described; the most common and best described exhibits physiologic airflow obstruction and is termed bronchiolitis obliterans syndrome (BOS). Unfortunately, regardless of the CLAD phenotype, by the time a clinical diagnosis is made, treatment is usually ineffective [2]. Earlier detection may improve treatment prospects, but there is currently no reliable method to detect CLAD before it is physiologically evident.

Many lung transplant centers utilize surveillance bronchoscopy with bronchoalveolar lavage (BAL) and transbronchial biopsy to monitor for asymptomatic acute rejection (AR) and infection. However, transbronchial biopsy is not a reliable method to diagnose CLAD due to the very small tissue size obtained and the patchy nature of the disease. However, BAL offers an alternative and larger window for observing lung biology as it samples a much larger area of the allograft. While the dilution factor may affect protein concentrations, this is not an issue when studying the cellular component returned in the BAL fluid. Therefore, transcription profiling of the BAL cell pellet (CP) may be a useful tool to monitor the immune response in the lung allograft and to provide mechanistic information about CLAD pathogenesis. Given that the onset of CLAD pathogenesis must precede our ability to make a clinical diagnosis, we hypothesized that transcription profiles from the BAL CP would be associated with incipient CLAD and be informative about the pathobiology responsible for CLAD development.

This study was conceptualized by Clinical Trials in Organ Transplantation (CTOT)-20 investigators in order to compile preliminary data for a Clinical Trials in Organ Transplantation (CTOT) ancillary studies proposal. CTOT-20 is a prospective multicenter observational cohort study to define the risk factors, mechanisms, and manifestations of CLAD phenotypes sponsored by the National Institute of Allergy and Infectious Diseases (NIAID). Samples were collected prior to initiation of CTOT-20 but consistent with the protocols and standards specified by CTOT. The protocol was approved by the UCLA Institutional Review Board (#10–001492) and all subjects provided written informed consent to participate in the study.

## Patients and Methods

### Identification of study patients

Lung transplant recipients at UCLA undergo surveillance bronchoscopy at 1, 3, 6, and 12 months post-transplant, and when clinically indicated. Since 2001, a subset of recipients was enrolled in an observational registry study that included the collection of BAL fluid for research purposes at the time of standard of care bronchoscopies. The registry includes standardized medical record abstraction including demographic, transplantation, and outcome related variables. For this nested case control study, eligible subjects were those with a 1 year surveillance bronchoscopy that was negative for rejection and infection, with the corresponding research BAL sample available in our biorepository. Subjects meeting these criteria were then screened for incipient CLAD and CLAD free phenotypes. Incipient CLAD was defined as a clinical diagnosis of CLAD within 730 days following the bronchoscopy. CLAD was diagnosed according to ISHLT criteria, defined as a sustained drop in FEV_1_ by at least 20% from the average of the 2 best post-transplant FEV_1_ measurements [3]. CLAD free control recipients remained without CLAD for at least 4 years following the 12 month bronchoscopy.

**Fig 1:**
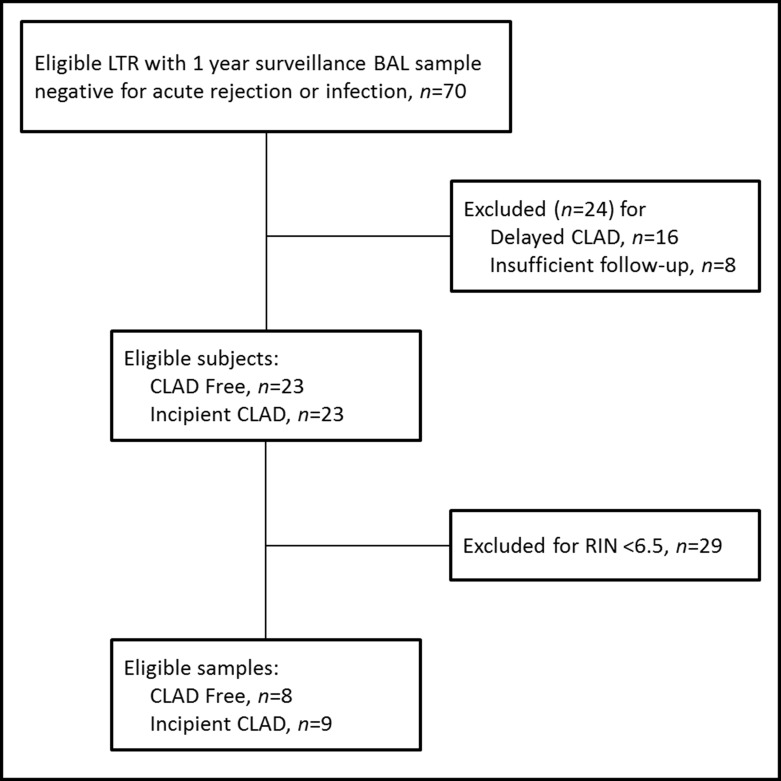
Sample selection flow chart. LTR, lung transplant recipient. BAL, bronchoalveolar lavage. CLAD, chronic lung allograft dysfunction. RIN, RNA integrity number.

Our repository included 70 BAL samples from eligible subjects, 23 which met criteria for incipient CLAD cases and 23 which met criteria for CLAD free controls (Fig 1). The remaining subjects were excluded for either delayed CLAD (n = 16) or insufficient follow-up time to establish freedom from CLAD for at least 4 years post-BAL (n = 8).

### Bronchoalveolar lavage fluid sample collection and processing

The BAL procedure was done according to a standardized protocol using three 60-ml aliquots of isotonic saline instilled into a subsegmental bronchus of either the right middle lobe or left lingula. Retrieved BAL fluid was pooled and then split into a 15 ml clinical specimen and a research specimen with the remaining volume. The freshly acquired research samples were immediately placed on ice for transport to the lab where they were processed within 6 hours of collection. Briefly, the BAL fluid was filtered through sterile gauze, the cells were counted, and cytospin preparations were made for differential cell counts. The remaining cells were separated from fluid by centrifugation. Cells were washed twice with phosphate-buffered saline and lysed in TRIzol (Invitrogen, Carlsbad, CA).

### RNA extraction and microarray analysis

Total RNA was isolated using TRIzol/chloroform extraction, re-suspended in RNase-free water and purified using the miRNeasy Mini kit (Qiagen Inc, Valencia, CA). RNA was discarded if the 260/280 ratio was not between 1.8 and 2.1, or if RNA showed evidence of degradation (RNA integrity number less than 7.0) when assessed with the Agilent 2100 BioAnalyzer (Agilent Technologies, Palo Alto, CA). In total, we were left with 9 incipient CLAD samples and 8 CLAD free samples of sufficient quality to move forward for microarray analysis.

The poly(A) RNA was converted to cDNA, which was used for in vitro transcription of biotin-labeled cRNA. Hybridization with the biotin labeled RNA, staining, and scanning of the chips followed the procedure outlined in the Affymetrix technical manual. All analyses used the Affymetrix Human U133 plus 2.0 array, which contains approximately 48,000 probe sets designed from GenBank, dbEST, and RefSeq sequences clustered based on build 133 of the UniGene database and an additional 6500 transcripts identified from Unigene build 159. Background correction utilized the Robust Multi-Array Average (RMA) method [4, 5]. Data were normalized with quantile normalization and Tukey’s Median Polish Approach was used to summarize probe intensities [6]. We focused on probe sets meeting the following criteria: 1) More than 50% arrays have expression index (log2 scale) of at least 3. This step eliminates probes with low expression index. 2) Coefficient of variation is greater than 0.2 across all arrays. This step excludes probes with low variability. Using these criteria, 23,271 out of 54,675 probes remained after non-specific filtering.

### Statistical methods

Subject’s clinical characteristics were displayed as a column percentage, mean ± SD, or median with interquartile range as described. Categorical data were compared using Fisher’s exact test. Continuous data were compared using the Mann-Whitney test.

Bioconductor package LIMMA (linear models for microarray data) [7] was used for differential gene expression analysis. Due to the relatively small number of arrays, empirical Bayesian method is adopted to provide stable testing results. A candidate list of differential expressed probe sets were identified using a volcano plot that showed > 2 fold or < -2 fold differential expression and were significant by LIMMA's moderated t-test (p < 0.05) between incipient CLAD and CLAD free. For functional annotation and pathway enrichment analysis, the candidate probes were analyzed in Database for Annotation, Visualization and Integrated Discovery (DAVID) [8, 9] and processes and pathways were selected based on Benjamini and Hochberg [10] adjusted p-values smaller than 0.05. Principal component analysis (PCA) [11] was used to visualize the separation of the two groups based on expression profiles of the selected candidate probes. Further unsupervised hierarchical clustering of differentially expressed probes was done by applying the Ward's minimum variance criterion linkage method [12] with Euclidean distance and presented in a heat map.

A supervised machine learning tool, support vector machine (SVM) [13], was used for classification of patient samples into incipient CLAD and CLAD-free categories. Essentially, a binary support vector machine is an algorithm that looks for the optimal hyperplane of separating the two classes by maximizing the margin between the closest points of the two classes. The points on the boundaries are called support vectors, while our optimal separating hyperplane is located in the middle of the margin. Literature suggests SVM classifiers have superior and robust performance in identifying predictive biomarkers in the setting of high-dimensional microarray gene expression data [14–18]. To overcome overfitting due to small number of arrays and large number of features, we also performed recursive feature elimination (SVM-RFE) algorithm [19] on the SVM to remove features with smallest ranking criterion, which corresponds to components of the SVM weight vector that are smallest in absolute value. Performance of our SVM is assessed by leave-one-out cross validation. All the statistical analyses were conducted using Bioconductor suite of packages [20] in the R statistical software environment version 3.2.3 [21].

The data discussed in this publication are available in the ArrayExpress database (http://www.ebi.ac.uk/arrayexpress) under accession number E-MTAB-5029 [22].

## Results

### Patient characteristics

Our repository included 23 samples which met criteria for incipient CLAD cases and 23 which met criteria for CLAD free controls (Fig 1). A priori, we specified we would include only high integrity RNA samples (RIN ≥6.5), thus we had to exclude 29 additional samples with RIN <6.5, leaving seventeen samples from unique subjects, including 9 samples with incipient CLAD and 8 samples classified as CLAD free. Subject characteristics of included and excluded subjects, for cases and controls, were similar between groups as shown in Table 1. All 9 included cases of incipient CLAD were consistent with the BOS phenotype, based upon stable FVC and lack of fibrotic changes on CT, as compared to 3 excluded subjects with restrictive CLAD. Kaplan Meier 1-, 3-, and 5-year survival estimates following a diagnosis of CLAD were 56%, 44%, and 22% and 57%, 36%, and 14% for included and excluded CLAD cases respectively (log-rank P = 0.72).

**Table 1:** Clinical characteristics of the CLAD Free and Incipient CLAD cohorts.

	CLAD Free Included (n = 8)	CLAD Free Excluded (n = 15)	Incipient CLAD Included (n = 9)	Incipient CLAD Excluded (n = 14)	*P*-value
Sex (%)					0.52
Male	3 (38)	10 (67)	6 (67)	9 (64)	
Female	5 (62)	5 (33)	3 (33)	5 (36)	
Age at transplant, mean ± SD	57.3 ± 9.7	54.2 ± 9.9	57.9 ± 6.6	60.7 ± 8.3	0.28
Pre-transplant disease (%)					0.95
COPD	3 (38)	5 (33)	2 (22)	5 (36)	
Restrictive lung disease	4 (50)	8 (53)	6 (67)	6 (43)	
Other	1 (12)	2 (13)	1 (11)	3 (21)	
Type of Transplant (%)					0.08
Bilateral	6 (75)	11 (73)	9 (100)	7 (50)	
Single	2 (25)	4 (37)	0 (0)	7 (50)	
FEV1% predicted, median (IQR)^†^	74 (68–98)	71 (51–93)	74 (72–91)	67 (57–89)	0.54
FEV1% of baseline, median (IQR)^†^	98 (96–100)	96 (93–100)	87 (84–95)	91 (86–96)	0.006^a^
Months to Sample, median (IQR)	12.4 (12.2–12.5)	12.6 (11.7–14.3)	13.6 (12.3–18.8)	12.8 (12.4–15.2)	0.24
RNA Integrity Number, median (IQR)	7.8 (7.1–8.0)	4.3 (2.7–5.5)	8.1 (7.6–9.0)	4.9 (3.3–5.2)	<0.0001^b^

^†^Characteristics are at the time of sample acquisition.

^a^Comparison of CLAD Free Included vs. Incipient CLAD Included by Mann Whitney *P* = 0.01.

^b^Comparison of CLAD Free Included vs. Incipient CLAD Included by Mann Whitney *P* = 0.14.

Among included samples, most characteristics of cases and controls were similar. While the FEV1 percent predicted were identical in CLAD free controls and incipient CLAD cases, the FEV1 percent of baseline was lower in the incipient CLAD group (P = 0.01), where 5 subjects met criteria for BOS0p as compared to no patient in the CLAD free cohort. At the time of sample collection, all includes cases and controls were on maintenance immune suppression consisting of tacrolimus, MMF, and low dose prednisone, except for 1 patient in each group where sirolimus had been exchanged for MMF. No patient was being treated with Azithromycin at the time of sample collection. In the CLAD free controls, the median time from sample to last follow-up was 78.4 (IQR 55.2–83.5) months. In the incipient CLAD cases, the time from sample to CLAD diagnosis was usually less than 1 year, with a median of 7.3 (IQR 3.3–12.9) months.

### BAL cell counts and differential

**Fig 2:**
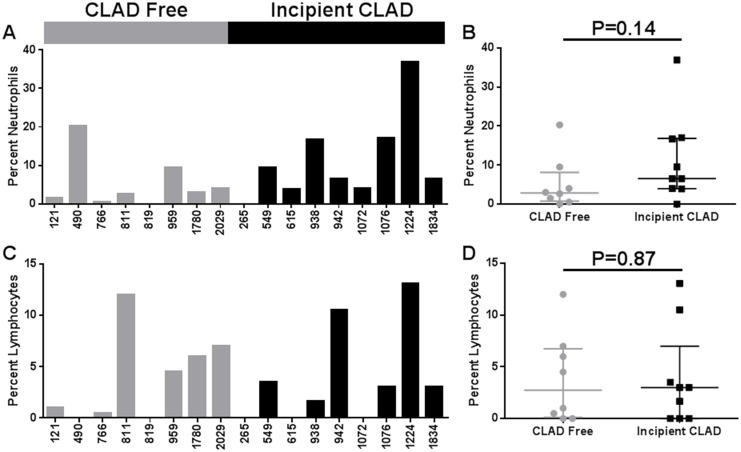
BAL cell differential. (A) Percentage of neutrophils among the BAL cells by sample. (B) Percent neutrophils analysis (Mann Whitney test) by CLAD Free versus Incipient CLAD group. (C) Percentage of lymphocytes among the BAL cells by sample. (D) Percent lymphocytes analysis (Mann Whitney test) by CLAD Free versus Incipient CLAD group.

We did not make any effort to fractionate cells before RNA isolation. Thus, it is possible that differences in gene expression could reflect differences in the leukocyte cellular proportions within the BAL CPs. To assess this, we compared the BAL total cell counts and differential counts between samples from CLAD free and incipient CLAD cases. The median total cell counts per ml of BAL fluid were similar in the CLAD free and incipient CLAD groups (1.1 vs. 1.4 x 10^5^, P = 0.62). Macrophages made the majority of cells for each sample. Neither neutrophil nor lymphocyte percentages differed significantly between CLAD free and incipient CLAD groups (Fig 2).

### Differential gene expression analyses

**Fig 3:**
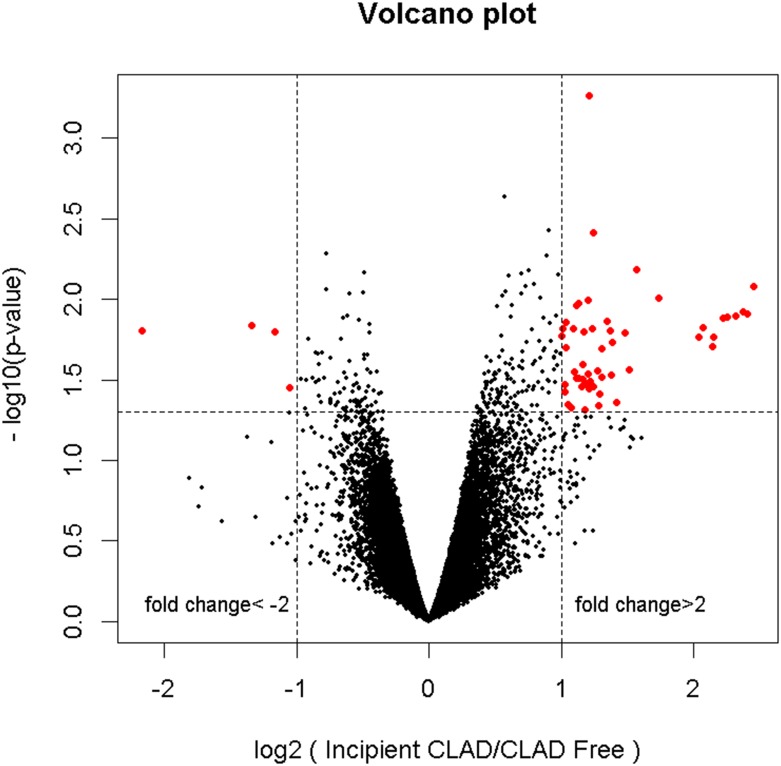
Volcano plot visualization of differential gene expression. DAE generated a candidate list of 55 probe sets, 51 over- and 4 under-expressed in the incipient CLAD as compared to the CLAD free group.

Based upon an absolute fold change >2.0 and unadjusted p-value <0.05, differential gene expression analysis identified 55 differentially expressed probe sets, 51 over- and 4 under-expressed probes with incipient CLAD as compared to the CLAD free group (Fig 3). The 55 differentially expressed probes map to 40 unique candidate genes, listed in Table 2.

**Table 2:** Unique genes differentially expressed during Incipient CLAD vs. No CLAD.

Gene Name	Gene Symbol (GenBank Acc)	FC	P-value
***Down-regulated genes***			
---	(BG389789)	-4.51	0.017
egf-like module containing, mucin-like, hormone receptor-like 1	EMR1	-2.53	0.015
---	(H78083)	-2.24	0.017
fibronectin type III domain containing 3B	FNDC3B	-2.07	0.037
***Up-regulated genes***			
chemokine (C-X-C motif) ligand 13	CXCL13	5.48	0.009
hemoglobin, alpha 1 /// hemoglobin, alpha 2	HBA1 /// HBA2	5.31	0.014
hemoglobin, beta	HBB	4.41	0.021
granzyme A (granzyme 1, cytotoxic T-lymphocyte-associated serine esterase 3)	GZMA	3.33	0.011
granzyme H (cathepsin G-like 2, protein h-CCPX)	GZMH	2.96	0.007
T cell receptor beta constant 1	TRBC1	2.86	0.029
B-cell CLL/lymphoma 11B (zinc finger protein)	BCL11B	2.79	0.017
chemokine (C-C motif) ligand 5	CCL5	2.67	0.046
CD8a molecule	CD8A	2.58	0.017
perforin 1 (pore forming protein)	PRF1	2.55	0.014
regulator of G-protein signaling 1	RGS1	2.48	0.021
LCK proto-oncogene, Src family tyrosine kinase	LCK	2.47	0.032
granulysin	GNLY	2.45	0.041
NLR family, CARD domain containing 3	NLRC3	2.42	0.029
---	(AI949912)	2.37	0.004
HOP homeobox	HOPX	2.35	0.016
ADP-ribosylation factor-like 4C	ARL4C	2.33	0.034
killer cell lectin-like receptor subfamily C, member 1, member 2	KLRC1 /// KLRC2	2.32	0.038
microRNA 6883 /// period circadian clock 1	PER1	2.31	0.001
killer cell lectin-like receptor subfamily K, member 1	KLRK1	2.31	0.031
eomesodermin	EOMES	2.30	0.010
natural killer cell granule protein 7	NKG7	2.29	0.036
---	(AI475680)	2.25	0.017
tripartite motif containing 58	TRIM58	2.24	0.033
annexin A1	ANXA1	2.23	0.036
B-cell translocation gene 1, anti-proliferative	BTG1	2.19	0.011
killer cell lectin-like receptor subfamily D, member 1	KLRD1	2.17	0.011
plastin 3	PLS3	2.14	0.029
---	RP11-489E7.4	2.13	0.016
ATPase, aminophospholipid transporter, class I, type 8B, member 2	ATP8B2	2.11	0.049
cytotoxic T-lymphocyte-associated protein 4	CTLA4	2.08	0.047
synuclein, alpha (non A4 component of amyloid precursor)	SNCA	2.05	0.014
chloride intracellular channel 3	CLIC3	2.05	0.020
killer cell lectin-like receptor subfamily B, member 1	KLRB1	2.04	0.035
transmembrane protein 200A	TMEM200A	2.04	0.039
sterile alpha motif domain containing 3	SAMD3	2.01	0.017

### Functional annotation and pathway enrichment analyses

The list of 40 candidate genes was submitted for functional annotation and pathway analysis using DAVID Bioinformatics Resources [9, 23]. Comparison of the biological process category of gene ontology (GO) classification indicated that the predominant processes were related to activation or differentiation of immune cells or to an immune response in general (Table 3). Similarly, pathways significantly enriched in this gene list included the Reactome pathway “signaling in immune system” and Kyoto Encyclopedia of Genes and Genomes (KEGG) pathways “natural killer cell mediated cytotoxicity” and “graft-versus-host disease” (Table 4).

**Table 3:** Gene ontology: biological processes significantly associated with incipient CLAD.

GOTERM	Count	%	Gene Symbols	P-Value	Adjusted P-Value
leukocyte activation	7	19.4	NLRC3, EOMES, SNCA, BCL11B, CD8A, LCK, KLRK1	1.28E-05	0.008
cell activation	7	19.4	NLRC3, EOMES, SNCA, BCL11B, CD8A, LCK, KLRK1	3.35–05	0.010
lymphocyte activation	6	16.7	NLRC3, EOMES, BCL11B, CD8A, LCK, KLRK1	6.92E-05	0.014
Immune response	9	25.0	CXCL13, EOMES, CCL5, SNCA, RGS1, CD8A, CTLA4, GZMA, TRBC1	9.46E-05	0.014
T cell activation	5	13.9	NLRC3, EOMES, BCL11B, CD8A, LCK	1.63E-04	0.019
T cell differentiation	4	11.1	EOMES, BCL11B, CD8A, LCK	3.92E-04	0.038

**Table 4:** Enriched Pathways among Differentially Expressed Genes in Incipient CLAD.

Category	Term	Count	%	Genes	p-value	adjusted p-value
Reactome Pathway	Signaling in Immune system	6	16.7	CD8a molecule; T cell receptor beta variable 19; Killer cell lectin-like receptor subfamily C, member 1; Killer cell lectin-like receptor subfamily D, member 1; Killer cell lectin-like receptor subfamily K, member 1; Lymphocyte-specific protein tyrosine kinase	2.3E-05	9.1E-05
KEGG Pathway	Natural killer cell mediated cytotoxicity	5	13.9	Killer cell lectin-like receptor subfamily C, member 1; Killer cell lectin-like receptor subfamily D, member 1; Killer cell lectin-like receptor subfamily K, member 1; Lymphocyte-specific protein tyrosine kinase; Perforin 1 (pore forming protein);	1.3E-04	0.0028
KEGG Pathway	Graft-versus-host disease	3	8.3	Killer cell lectin-like receptor subfamily D, member 1; Killer cell lectin-like receptor subfamily K, member 1; Perforin 1 (pore forming protein)	0.0030	0.033
KEGG Pathway	Antigen processing and presentation	3	8.3	CD8a molecule; Killer cell lectin-like receptor subfamily C, member 1; Killer cell lectin-like receptor subfamily D, member 1	0.013	0.093
KEGG Pathway	T cell receptor signaling pathway	3	8.1	CD8a molecule; Cytotoxic T-lymphocyte-associated protein 4; Lymphocyte-specific protein tyrosine kinase	0.022	0.11

### Cluster analysis of significant genes and relationship to incipient CLAD

**Fig 4:**
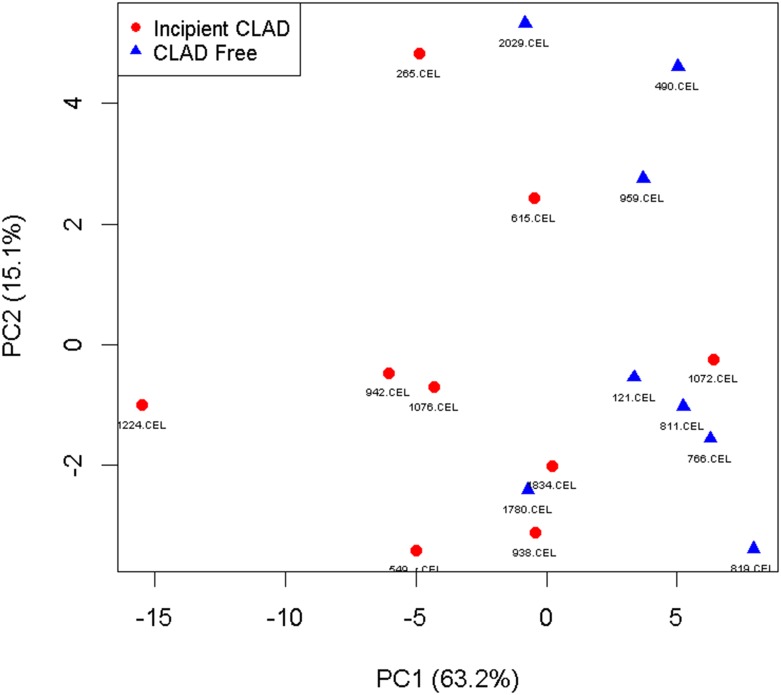
Principal component analysis. Principal component analysis based on the 40 differentially expressed candidate genes demonstrates modest separation of incipient CLAD and CLAD free groups. The percentage of total variance accounted for by the first principal component was 63.2%, and for the second principal component was 15.1%.

**Fig 5:**
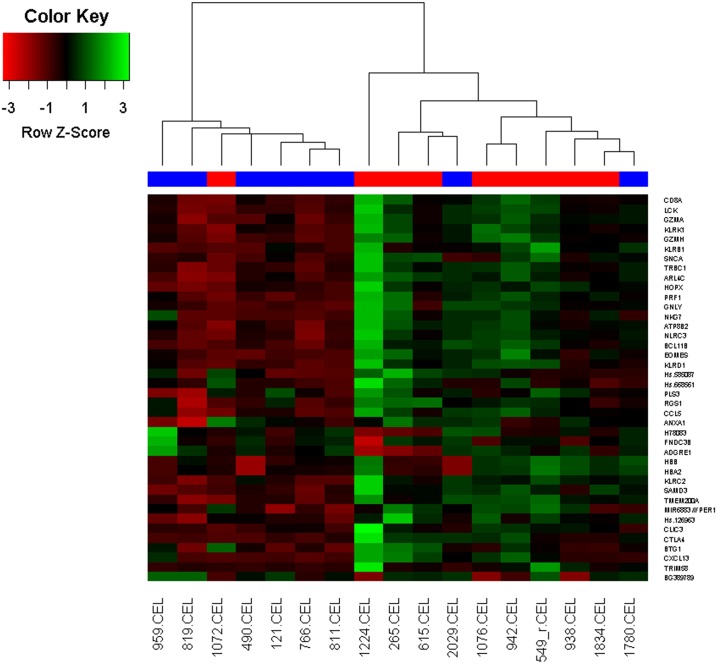
Hierarchical clustering and heat map. Hierarchical clustering and heat map based on the expression index of n = 40 candidate genes.

Principle Component Analysis based on the 40 unique genes differentially expressed demonstrated modest separation of incipient CLAD and CLAD free samples (Fig 4). To further evaluate the potential for our gene list to discriminate incipient CLAD patients from no CLAD patients, we performed unsupervised hierarchical clustering based on the Euclidean distance dissimilarity measure, and Ward’s minimum variance criterion linkage method. Using these methods, we identified 2 major clusters of patient samples. In the first cluster, 6 of 7 samples were from no CLAD patients. In the second cluster, 8 of 10 samples were from incipient CLAD patients (Fig 5).

### Classifier training, feature selection and cross validation

We developed a support vector machine (SVM) classifier to separate patient samples into incipient CLAD and CLAD-free categories. The training data set consists of 40 features from all 17 arrays (9 incipient CLAD, 8 CLAD-free). Our SVM is trained by both linear and non-linear kernel functions with a varying set of kernel parameters. Based on 40 features, a SVM with linear kernel and cost function = 1 is constructed. There are 7 support vectors (i.e. 7 arrays on the boundaries). The leave-one-out cross validation error is 11.7%. This SVM correctly classifies 14 out of the 17 samples with accuracy of 82.3%. After recursive feature elimination, a SVM with the top 10 features has a 94.1% accuracy (16 out of 17 correctly identified) on the training data based on leave-one-out cross validation.

## Discussion

This study of the BAL CP transcriptome after lung transplantation is the first to examine gene expression relative to the future development of CLAD. We make the novel observation that incipient CLAD, defined as CLAD onset within 2 years of BAL collection, is associated with a distinct BAL CP gene expression profile. More specifically, the incipient CLAD profile predominantly exhibits differentially expressed genes related to recruitment, retention, activation and proliferation of cytotoxic lymphocytes (CD8^+^ T-cells and natural killer [NK] cells). These findings validate the utility of the BAL CP transcriptome as a tool for investigating the pathogenesis of CLAD. Furthermore, we confirm that the onset of pathogenesis precedes our ability to make the clinical diagnosis of CLAD, and suggests that the BAL CP transcriptome is a useful biomarker for CLAD development.

While we note a non-significant trend for increased percentage of BAL neutrophils in incipient CLAD samples, the differentially expressed gene profile was not suggestive of a neutrophil mediated process. In contrast, several differentially expressed genes favor recruitment and retention of mononuclear immune cells in the lung allograft that develops CLAD. For instance, incipient CLAD is associated with upregulated expression of the CC chemokine, RANTES/CCL5, a chemoattractant for mononuclear leukocytes [24]. Importantly, we have previously found CCL5 protein concentrations to be increased in human BALF during both AR and pulmonary cytomegalovirus (CMV) infection, both putative risk factors for CLAD [24, 25]. Furthermore, in rodent models, we and others have demonstrated that in vivo neutralization of CCL5 significantly attenuates lung and airway allograft rejection [24]. We also find that incipient CLAD is associated with the upregulated expression of CXCL13. CXCL13 is most commonly known to recruit and control the organization of B cells within lymphoid follicles [26]. However, CXCL13 expression is also induced in mature macrophages during chronic inflammation and CXCL13 expression by lymphoid like stroma within the allograft has been described in renal and cardiac transplant rejection, as well as with CLAD [27–29].

In addition to genes related to the recruitment and retention of mononuclear immune cells in incipient CLAD samples, we also find differential gene expression related to activation and proliferation of these cells. In particular, prior to the development of CLAD we find differential expression of genes related to activation and proliferation of cytotoxic lymphocytes (CD8^+^ T-cells and NK cells). For instance, we find increased expression of both CD8a and LCK proto-oncogene, Src family tyrosine kinase (LCK). CD8 acts to stabilize binding of the T-cell receptor (TCR) to the peptide-MHC complex, while also localizing LCK to the TCR/CD3 complex to facilitate early signaling events during T-cell activation [30]. Furthermore, incipient CLAD was associated with upregulated expression of genes in the NKG2 family of receptors (killer cell lectin like receptor [KLR] C1, KLRD1, KLRK1), which are expressed by both NK and CD8^+^ cells and provide activating or costimulatory signals [31]. Similarly, KLRB1 was upregulated in incipient CLAD samples, which is primarily thought to mediate NK cell function [32]. We also find increased expression of transcripts encoding key regulators of cytotoxic cells including regulator of G Protein Signaling-1 (RGS1), eomesodermin (EOMES), B-cell CLL/lymphoma 11B (zinc finger protein), synuclein, alpha (SNCA), and T cell receptor beta constant 1 (TRBC1), all of which are involved in different aspects of differentiation and proliferation/clonal expansion of effector memory cells [33–37]. Finally, both CD8^+^ T cells and NK cells have conserved pathways that mediate target allograft cell injury via perforin, granzymes and granulysin, each of which is differentially over-expressed in incipient CLAD cases [38].

It is also of interest that multiple genes related to hemoglobin (HB) expression are increased in the BAL CP from cases of incipient CLAD. Although RBC’s lack a nucleus, a microarray study of human RBC’s demonstrated the presence of transcripts for 1019 genes in human RBCs including genes for hemoglobin [39]. Therefore, we have interpreted the evidence of hemoglobin transcripts in the BAL CP as evidence for RBC’s in the BAL CP. Taken in the context with simultaneous upregulated signals for potentially injurious CD8^+^ T cells and NK cells, this suggests a subclinical lung allograft injury with micro-hemorrhage. Importantly, we have also found that incipient CLAD CPs have a distinct downregulation of fibronectin type III domain containing 3B (FNDC3B), which is considered a putative marker of stem/progenitor cells [40]. This derangement may indicate an inability for allograft lung to repair and reverse remodel allowing for structural damage and CLAD.

Functional annotation analyses of our list of differentially expressed genes in incipient CLAD samples led to similar conclusions. Enriched GO terms suggest activation and differentiation of mononuclear immune cells. Similarly, our gene list was significantly enriched for several biologic pathways, each related to either innate or adaptive immunity. Taken together, these findings indicate that the pathogenesis of CLAD involves immune activation that would be expected with a rejection process, and that these biologic derangements are evident in the BAL CP prior to our ability to make a clinical diagnosis of CLAD.

In fact, our study suggests that the performance of the BAL CP transcriptome as a biomarker for predicting CLAD risk may be quite good. Hierarchical cluster analyses correctly grouped the majority of incipient CLAD and CLAD free samples. In these analyses, one incipient CLAD sample (#1072) was misclassified with CLAD free samples. In this case, the CLAD diagnosis occurred 167 days after sampling, concurrent with a subtherapeutic tacrolimus trough, after previous levels were consistently within or above the target range. Two CLAD free samples (#1780 and #2029) were misclassified with incipient CLAD samples. These patients remained CLAD free at the end of follow-up 1399 and 1568 days after the respective sample dates. The first subject (#1780) had previously experienced refractory AR, which was treated with basiliximab and IVIG, with resolution of AR by the time of the study sample. Thereafter, the patient’s immune suppressive regimen was changed from Tacrolimus/MMF/prednisone to Tacrolimus/Rapamycin/prednisone due to concern about elevated risk of CLAD. The second subject (#2029) experienced a 12% decline in FEV1 shortly after the study sample. Azithromycin immunomodulation (250 mg M,W,F) was added to the patient’s immune suppression regimen. It is not possible to know whether the changes in management impacted the allograft immunobiology and CLAD outcomes in these 2 patients.

Our study is the first to examine BAL CP gene expression patterns relative to CLAD pathogenesis. However, there are strikingly common themes in several previous studies of gene expression in the lung during biopsy proven AR. Two such studies have examined BAL CP gene expression during AR and concluded that the BAL CP is a reliable source of RNA for transcriptome analysis [41, 42]. Similar to our findings with incipient CLAD, AR was associated with differential expression of granzyme, perforin, CD8, cytotoxic T-lymphocyte-associated protein 4 (CTLA-4), and T-cell receptor genes. Our group has also recently published RNA-Seq gene expression profiles from BAL fluid exosomes in lung transplant recipients with AR, and contrasted exosomal profiles with the CP from the same BAL sample [43]. In that study, the AR BAL CP expression profiles again exhibit similarities to incipient CLAD samples. Specific genes we find upregulated from both AR and incipient CLAD CPs included CXCL13, CTLA-4, and KLRC1 [43]. Given the similarities in gene expression, it is possible that incipient CLAD is synonymous with undiagnosed AR, and CLAD may represent the consequence of untreated subclinical AR. Previous studies suggest that even a single episode of minimal AR places the patient at increased risk for CLAD [44, 45]. Although transbronchial biopsy is the current “gold standard” for AR diagnosis, the sampling error and the interobserver variability of biopsy interpretation are well documented [42, 46–49]. Furthermore, transbronchial biopsy is invasive and associated with risks of bleeding, pneumothorax, and respiratory failure. Therefore, the lower risk and larger area of lung sampled by BAL, as well as the lack of subjectivity involved with gene expression analysis, may be advantages over transbronchial biopsy for monitoring allograft status. As acute cellular rejection is defined based on alveolar infiltration while CLAD (BOS phenotype) is airway centric, perhaps CLAD and AR both represent allorecognition manifest as a specific T cell signature, and that perhaps CLAD and AR differ in target antigen and compartment (bronchiole in CLAD vs alveolus in AR). If future studies confirm gene expression similarities between AR and incipient CLAD, then perhaps augmented immune suppression could prevent progression to CLAD in these at-risk patients. The prospect of a better, safer test to guide therapies which may prevent CLAD could have enormous implications on the lung transplant field. However, given our small sample size and lack of a controlled intervention, these findings require confirmation in a larger multicenter study, followed by an interventional clinical trial, before they can impact our practice.

In addition to our small sample size, other factors in this study warrant caution in interpreting our findings. We defined CLAD as a 20% decline in FEV1 from baseline and excluded samples collected after CLAD. “Potential CLAD”, which can be defined as a 10% decline in FEV1 from baseline, was not considered CLAD. All 5 patients with “potential CLAD” ended up in the incipient CLAD group. We explored repeating the analyses after exclusion of patients in the potential-CLAD group and our major findings and interpretations were not affected (data not shown). The very specific selection criteria for samples used in this study may limit the generalizability of our findings. Our incipient CLAD cases went on to develop CLAD within 2 years of sampling. Our CLAD free controls were patients who remained CLAD free for the duration of follow-up and for at least 4 years following the BALF sample. Patients who develop CLAD within 2–4 years were excluded from this study and it is unknown what expression profile this group would exhibit. We also excluded patient samples with either concurrent infection or AR. While this allowed for the focused analyses of the differentially expressed genes associated with incipient CLAD, the application of these methods in the clinic would be complicated by these relatively common occurrences in patients. The design of future studies would benefit from a larger sample size and inclusion of a more representative group of specimens.

In summary, we showed that the BAL CP transcriptome in incipient CLAD cases was indicative of activated innate and adaptive cytotoxic immune responses. These findings indicate that a pathobiology, similar to AR, precedes a clinical diagnosis of CLAD. Both hierarchical clustering and a supervised machine learning tool were able to correctly categorize most samples into incipient CLAD and CLAD-free categories, suggesting potential prognostic utility. A larger prospective investigation of BAL CP transcriptome as a biomarker for CLAD risk stratification is warranted.
